# Identification and Functional Analysis of an Epsilon Class Glutathione S-Transferase Gene Associated with α-Pinene Adaptation in *Monochamus alternatus*

**DOI:** 10.3390/ijms242417376

**Published:** 2023-12-12

**Authors:** Mingyu Xue, Xiaohong Xia, Yadi Deng, Fei Teng, Shiyue Zhao, Hui Li, Dejun Hao, Wei-Yi Chen

**Affiliations:** 1 Co-Innovation Center for Sustainable Forestry in Southern China, College of Forestry, Nanjing Forestry University, Nanjing 210037, Chinadengyadi1@gmail.com (Y.D.); lihui@njfu.edu.cn (H.L.); 2 Soochow College, Soochow University, Suzhou 215006, China

**Keywords:** glutathione S-transferase, detoxification enzymes, monoterpene

## Abstract

Alpha-pinene is one of the main defensive components in conifers. *Monochamus alternatus* (Coleoptera: Cerambycidae), a wood borer feeding on Pinaceae plants, relies on its detoxifying enzymes to resist the defensive terpenoids. Here, we assayed the peroxide level and GST activity of *M. alternatus* larvae treated with different concentrations of α-pinene. Meanwhile, a *gst* gene (*MaGSTe3*) was isolated and analyzed. We determined its expression level and verified its function. The results showed that α-pinene treatment led to membrane lipid peroxidation and thus increased the GST activity. Expression of *MaGSTe3* was significantly upregulated in guts following exposure to α-pinene, which has a similar pattern with the malonaldehyde level. In vitro expression and disk diffusion assay showed that the MaGSTe3 protein had high antioxidant capacity. However, RNAi treatment of MaGSTe3 did not reduce the hydrogen peroxide and malonaldehyde levels, while GST activity was significantly reduced. These results suggested MaGSTe3 takes part in α-pinene adaptation, but it does not play a great role in the resistance of *M. alternatus* larvae to α-pinene.

## 1. Introduction

In the long evolutionary process, conifers have developed efficient methods to resist the invasion of herbivorous insects and pathogens [1]. The defensive systems of conifers include constitutive and induced defenses [2]. Constitutive defenses are comprehensive defenses based on the corkification of bark, resin secretion, and phenolic synthesis, while induced defenses are responses to specific attacks through the synthesis of secondary metabolites [3,4,5]. Meanwhile, insects have also developed several mechanisms to help them break through the defenses. Insects can reduce the toxicity of defensive compounds through metabolism induced by detoxification enzymes or decrease oxidative damage caused by the compounds through antioxidant enzymes [6,7].

Masson pine (*Pinus massoniana* Lamb) is an important species for afforestation and timber in China. It has a special terpenoid defense system that consists chiefly of α-pinene and β-pinene. Of the two, α-pinene exhibits stronger insecticidal activity than β-pinene [8]. A previous study has shown that α-pinene can disrupt energy metabolism by the uncoupling of oxidative phosphorylation and the inhibition of electron transfer [9]. Also, it can enhance the generation of reactive oxygen species (ROS), leading to oxidative stress in tissues [10]. As a result, it is believed that the defensive mechanism of α-pinene is associated with oxidative damage to insects [11,12].

In the past decades, *P. massoniana* forests in China have been devastated by the pine wood nematode (PWN, *Bursaphelenchus xylophilus*), which is the pathogen of pine wilt disease [13]. Usually, the PWN is carried by wood borers and infects host plants through wounds caused by their feeding behavior [14]. In China, its primary vector is *Monochamus alternatus*, whose preferred host is *P. massoniana* [15]. Therefore, prevention and control of *M. alternatus* have attracted increasing attention. Usually, *M. altrenatus* larvae feed on the xylem of the host, and their feeding behavior can significantly induce α-pinene synthesis in the phloem and xylem of *P. massoniana* [16]. As result, α-pinene stress is inevitable for them, and they must have methods to overcome the stress. A previous study showed that exposure to α-pinene caused differential expression of cytochrome P450 mono-oxygenases (P450s), uridine diphosphate glycosyltransferases (UGTs), glutathione S-transferases (GSTs), and ATP-binding cassettes (ABCs) in *M. altrenatus* larvae [17]. As GSTs have an important effect on oxidative stress resistance, they may also be involved in the resistance of *M. altrenatus* to α-pinene stress [18].

In this study, we measured the level of membrane lipid peroxidation of larvae under α-pinene stress. Meanwhile, we isolated and characterized a *gst* gene (*MaGSTe3*), which was the only *gst* gene significantly upregulated in the transcriptome data of our previous study [17], and studied its role in the α-pinene resistance of *M. altrenatus* larvae. Our results will help clarify the role of GSTs in the resistance of *M. altrenatus* to α-pinene and understand the adaptive mechanisms of *M. altrenatus* to toxic terpenes, thus laying a foundation for the development of its control strategy.

## 2. Results

### 2.1. The Effects of α-Pinene Treatment on M. alternatus Larvae

**Figure 1:**
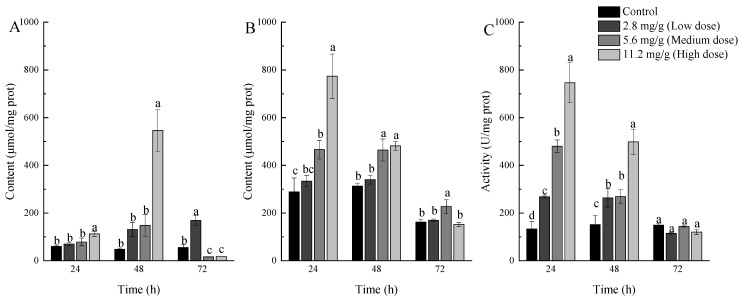
The contents of (**A**) hydrogen peroxide and (**B**) MDA, and (**C**) the GST activity of *M. alternatus* larvae at 24, 48, and 72 h after treatment with three different concentrations (2.8, 5.6, and 11.2 mg/g) of α-pinene. The contents of hydrogen peroxide and MDA were represented as μmol/mg protein. Data are presented as mean values ± standard deviation (SD). The differences between different groups were analyzed by one-way ANOVA with a significance level of *p* < 0.05, and different letters showed significant difference.

As is shown in Figure 1, the exposure to α-pinene caused hydrogen peroxide and MDA levels to increase in the medium- and high-dose groups after treatment for 24 and 48 h. In particular, the MDA levels rapidly reached 2.68- and 1.61-fold of the control, respectively, after treatment for 24 h and decreased to 1.54- and 1.48-fold of the control, respectively, 48 h after treatment (Figure 1B). 

Similarly, the GST activity was also increased in the high- and medium-dose groups, which reached 5.59- and 3.60-fold of the control at 24 h after treatment, respectively, and decreased to 3.29- and 1.78-fold of the control at 48 h, respectively, after treatment (Figure 1C). Interestingly, though there was no significant difference with the control in hydrogen peroxide and MDA content of the low-dose group, the group still had significantly higher GST activities at 24 and 48 h after treatment, which were 2.01- and 1.74-fold of the control, respectively. These results indicated that GSTs were involved in the adaptability of α-pinene stress.

### 2.2. Physicochemical Properties and Bioinformatics Analysis of MaGSTe3

The length of the *MaGSTe3* sequence is 648 bp, which encodes a protein with 215 amino acids with a predicted molecular weight of 23.58 kDa and a theoretical pI of 5.72. Subcellular localization shows that MaGSTe3 is in cytoplasm and has no transmembrane regions (Table A4). The 3D structure of MaGSTe3 shows that it contains 12 α-helices and 6 β-sheets (Figure A2), including a N-terminal domain with βαβαββα topology and a C-terminal domain. The conserved domain prediction shows that MaGSTe3 has multiple glutathione and substrate binding sites (Figure A3).

**Figure 2:**
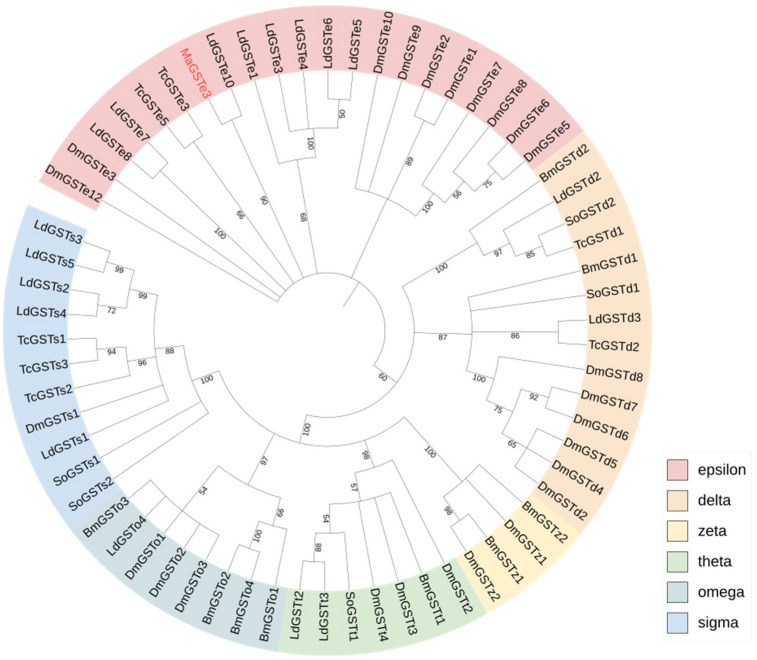
The phylogenetic tree of *MaGSTe3*. A total of 64 GST insect sequences from 5 species were used to construct the tree by the maximum likelihood (ML) method, with a total of 1000 bootstrap replications. The abbreviations of species are as follows: Dm—*Drosophila melanogaster*, Bm—*Bombyx mori*, Tc—*Tribolium castaneum*, Ld—*Leptinotarsa decemlineata*, and So—*Sitophilus oryzae*.

The results of BLASTx comparison showed MaGSTe3 had high homology with *Anoplophora glabripennis* (Table A4). The phylogenetic tree constructed with the maximum likelihood method presented the evolutionary relationship of MaGSTe3 (Figure 2). MaGSTe3 was clustered in the epsilon class with a *Leptinotarsa decemlineata* GST (LdGSTe10) in the same clade.

### 2.3. Response of MaGSTe3 to α-Pinene Stress

**Figure 3:**
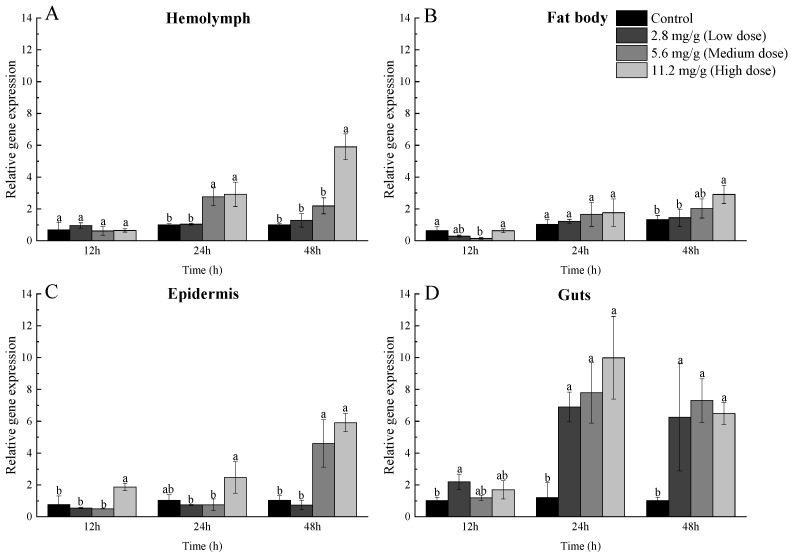
Relative expression level of *MaGSTe3* in (**A**) hemolymph, (**B**) fat body, (**C**) epidermis, and (**D**) guts at 12, 24, and 48 h after treatment with three different concentrations (2.8, 5.6, and 11.2 mg/g) of α-pinene. Data are presented as mean values ± SD. The differences between different groups were analyzed by one-way ANOVA with a significance level of *p* < 0.05, and different letters showed significant differences.

To further analyze the role of *MaGSTe3* in α-pinene adaptability, qRT-PCR was performed to estimate the response of *MaGSTe3* to α-pinene stress. The results showed that the relative expression of *MaGSTe3* was, overall, not changed at 12 h after treatment but remarkably upregulated at 24 h and 48 h after treatment (Figure 3). In particular, *MaGSTe3* was most highly upregulated in guts (Figure 3D). The transcriptional levels of low-, medium-, and high-dose groups was 5.76-, 6.51-, and 8.34-fold of control after treatment for 24 h, and 6.17-, 7.21-, and 6.41-fold of control after treatment for 48 h, respectively. Meanwhile, *MaGSTe3* showed significant upregulation in the high-dose group at 24 and 48 h after treatment in hemolymph and at all three time points in epidermis (Figure 3A,C).

### 2.4. Enzymatic Properties of Recombinant MaGSTe3

**Figure A1:**
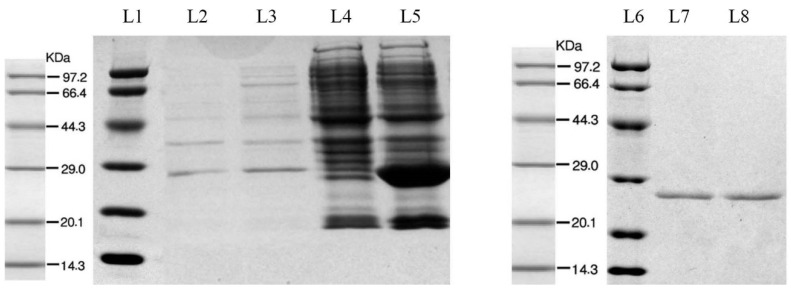
SDS-PAGE analysis of MaGSTe3 after recombinant expression and purification. L1 and L6 are protein molecular standards. L2 and L4 are the supernatant and precipitate, respectively, of the empty pET-28a (+) vector after 1 mM IPTG induction. L3 and L5 are the supernatant and precipitate, respectively, of pET-28a (+)-inserted MaGSTe3 after 1 mM IPTG induction. L7 and L8 are purified MaGSTe3.

**Figure A2:**
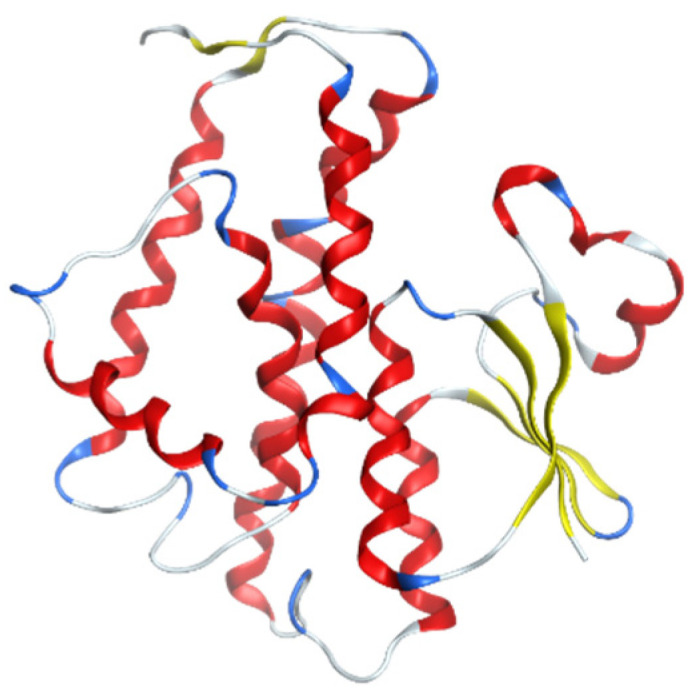
Three-dimensional structure of MaGSTe3 predicted by SWISS-MODEL. The red parts are α-helices and the yellow parts are β-sheets.

**Figure A3:**
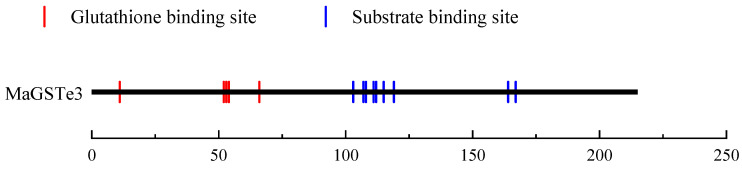
The conserved domain of MaGSTe3.

The *MaGSTe3* was amplified and inserted into pET-28a (+) and expressed in *E. coli* with IPTG induction. SDS-PAGE analysis (Figure A1) showed that protein bands with an approximate molecular weight of 28 kDa appeared in both precipitate and supernatant. The concentration of the MaGSTe3 solution obtained from purification was approximately 1 mg/mL.

**Figure 4:**
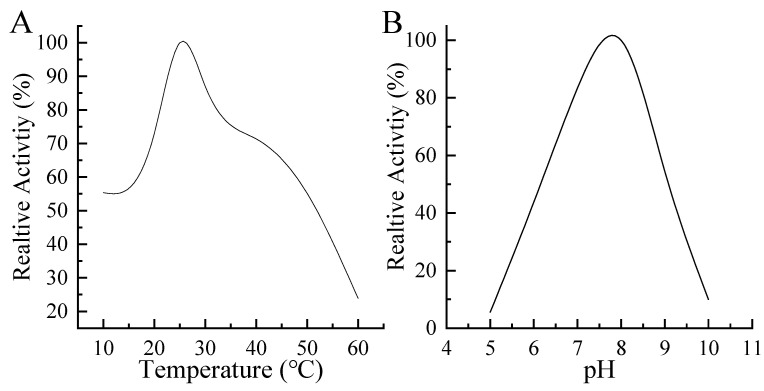
Enzymatic properties of recombinant MaGSTe3. (**A**) Relative activity of purified recombinant MaGSTe3 at different temperatures. (**B**) Relative activity of purified recombinant MaGSTe3 at various pH values.

The recombinant MaGSTe3 was incubated at a temperature range of 10 to 70 °C for 30 min, and the maximum activity was detected at 25 °C (Figure 4A). Also, the recombinant MaGSTe3 exhibited optimum catalytic activity at pH 7.7 (Figure 4B). The kinetic parameters determination showed that the Vmax and Km of recombinant MaGSTe3 were 2985.78 ± 347.15 μmol·min^−1^·mg^−1^ and 1.24 ± 0.21 mM, respectively. These results suggested that MaGSTe3 is catalytically active, displaying glutathione transferase activity.

### 2.5. Disk Diffusion Assay of MaGSTe3

**Figure 5:**
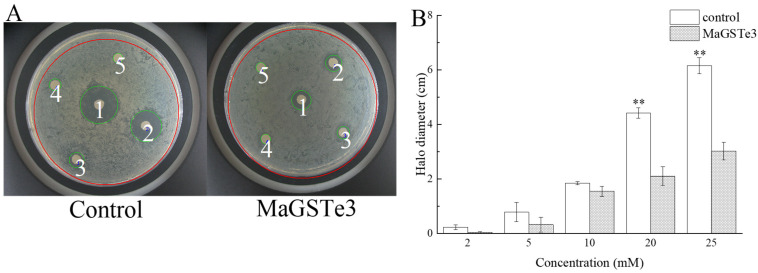
Disc diffusion assays using *E. coli* overexpressing *MaGSTe3*. (**A**) Resistance of *E. coli* cells overexpressing *MaGSTe3* to cumene hydroperoxide. The labels 1~5 on filter disks represent 25, 20, 10, 5, and 2 mM cumene hydroperoxide, respectively. (**B**) Histograms comparing halo diameters of inhibition zones. Data are presented as mean values ± SD. The differences between treatment groups and control were analyzed by Student’s *t* test, and asterisks mean significant differences: (**) *p* < 0.01.

Antioxidant activity of MaGSTe3 was detected by disk diffusion assay (Figure 5). *E. coli* cells carrying overexpressed MaGSTe3 were exposed to cumene hydroperoxide (CHP) to induce oxidative stress. After incubated for 24 h, the halo diameter of inhibition zones had no significant difference between treatment group and control when CHP level was 2–10 mM. Nevertheless, when the CHP level was increased to more than 20 mM, the halo diameter of inhibition zones could reduce more than 51% in the treatment group (Figure 5B).

### 2.6. RNAi of MaGSTe3

**Figure 6:**
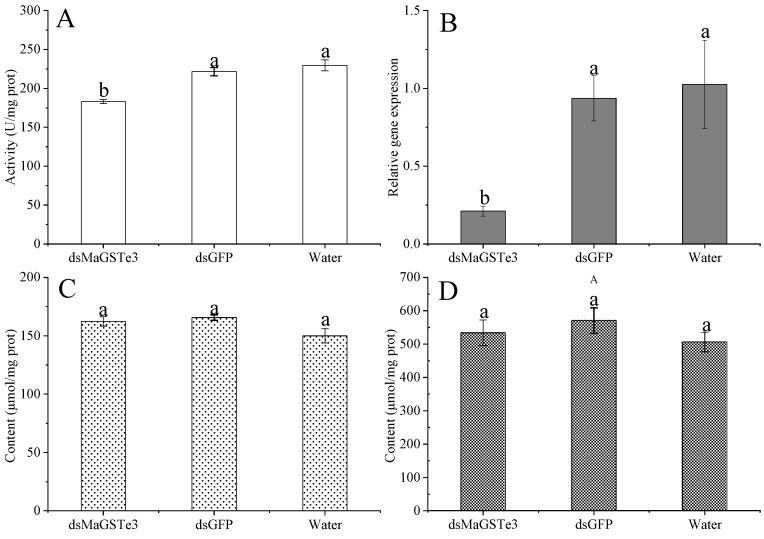
Effect of RNAi treatment on (**A**) GST activities, (**B**) relative expression level of *MaGSTe3*, (**C**) hydrogen peroxide, and (**D**) MDA levels. All larvae were treated with 5.6 mg·g^−1^ α-pinene. The contents of hydrogen peroxide and MDA were represented as μmol/mg protein. Data are presented as mean values ± SD. The differences between different groups were analyzed by one-way ANOVA with a significance level of *p* < 0.05, and different letters showed significant difference.

RNAi of *MaGSTe3* was performed to further verify the function of MaGSTe3. The dsRNA was injected into the intersegment membrane of *M. alternatus* and caused significant reduction in the GST activity and expression level of *MaGSTe3* (Figure 6A,B). However, the hydrogen peroxide and MDA levels did not show any differences (Figure 6C,D). These results indicated that there might be other enzymes involved in the antioxidation and detoxification of α-pinene stress.

## 3. Discussion

As the major defensive component in *P. massoniana* [19], α-pinene was believed to have strong biological activities against herbivorous insects [20]. A-pinene was observed to induce the antioxidant system in the Mediterranean flour moth, *Ephestia kuehniella* Zeller, and can inhibit feeding activity of the red turpentine beetle, *Dendroctonus valens* [7,21]. These phenomena suggest that oxidative damage may have occurred in the insect gut under the influence of α-pinene. As the main product of membrane peroxidation, the content of MDA can directly reflect the strength of oxidative damage [22]. In our study, the MDA level in the guts of *M. alternatus* larvae was rapidly increased after α-pinene treatment, and the content seems to be related to the concentration of α-pinene, suggesting that α-pinene might be the direct cause of the disorder in guts. Nevertheless, the MDA levels were decreased at 48 h after treatment and returned to normal at 72 h, which was similar with the trend of GST activity. Many studies have shown that oxidative stress induced by different factors all led to an increase in GST activity in insects [7,23,24]. Moreover, it was reported that GSTs participate in the reduction in lipid peroxides in *Drosophila melanogaster* [25]. As a result, GSTs may play roles in the adaptability of the α-pinene of *M. alternatus* by relieving the oxidative stress. 

GSTs in insects are divided into six families [26]. It is believed that GSTs from delta and epsilon families are crucial for insecticide resistance and the detoxification of plant xenobiotics [27,28,29,30]. In Coleoptera, epsilon GSTs might play an important role in detoxification. There are at least 12 out of 25 *DaGST*s found in the Chinese white pine beetle, *Dendroctonus armandi*, and 7 out of 14 *PtsGST*s in *Pagiophloeus tsushimanus*, which are reported to respond to the defensive components of host trees that are classified as being in the epsilon family [31,32,33]. In our study, *MaGSTe3* was significantly upregulated in guts when responding to α-pinene stress. Many studies have reported the abundant expression of *gst* genes in insect guts, especially in the midgut [29,34,35]. It is generally believed that the midgut is one of the tissues where insect GSTs mainly function, as it is the largest part of the digestive tract and the main target of plant defensive substances [36]. The role of MaGSTe3 in guts might be mediating oxidative stress responses. We also found that *MaGSTe3* in the high-dose treatment group was consistently upregulated in the epidermis. Several studies have also reported that volatile pesticide substances could lead to overexpression of *gst* in the epidermis [37,38]. However, the function of GSTs in other tissues is still unknown. Considering that plant terpenoids like α-pinene are volatiles that can be ingested by contact with the cuticle or fumigation, MaGSTe3 may play a part in cuticular resistance with similar functions in guts [37,39].

Disk diffusion assays and RNAi treatment were performed for function verification of MaGSTe3. Smaller inhibition zones in overexpressed groups than in control demonstrated the antioxidant capacity of MaGSTe3. Meanwhile, RNAi treatment of *MaGSTe3* significantly reduced the GST activity of *M. alternatus* larvae. However, RNAi treatment of *MaGSTe3* did not reduce the level of hydrogen peroxide and MDA. Many studies have shown that resistance to oxidative stress based on GSTs usually involves multiple enzymes from the same or different GST classes [24,31,32]. Though *MaGSTe3* is the only *gst* gene detected in the transcriptome of our study, we cannot deny the possibility of other GSTs participating. For example, GST genes from the sigma and omega classes, though their functions are not yet clear, were also observed to be upregulated when responding to oxidative stress, but their expression level is relatively low [31,40,41,42]. Moreover, epsilon GSTs were reported differentially regulated in the different feeding and terpenoids treatments [31,32]. There is a possibility that other *gst* genes, which were not originally upregulated, may have a substitute effect in *M. alternatus* larvae when *MaGSTe3* was knocked down. Thus, further study on the expression profiling of *MaGST*s is needed to clarify the role of GSTs in the α-pinene adaptation of *M. alternatus*. Another possibility is that MaGSTs do not play a major role in α-pinene resistance. Our previous study found that P450s and UGTs were the two genes with the highest number of upregulated expressions in detoxification enzymes, and knockdown of P450s increased mortality in the larvae under α-pinene stress [17]. Some studies reported that the P450 gene has specific expansion in conifer pests such as *Dendrolimus punctatus*, *Thaumetopoea pityocampa*, and *D. ponderosae*, indicating that P450s may be the key factor in resistance to host defensive substances [43,44]. It was also observed that UGTs are associated with eliminating oxidative stress [45]. Therefore, *MaGSTe3* may play a supporting role in the detoxification process of P450, and knockdown of *MaGSTe3* will not affect the detoxification of α-pinene in this case. However, the relationship between the P450s and GSTs of *M. alternatus* still needs further research.

## 4. Materials and Methods

### 4.1. Insect Collection and Treatment

The 2nd- and 3rd-instar *M. alternatus* larvae were collected from diseased and dead pines in Jiujiang city (115°56′35″ E, 29°34′1″ N), Jiangxi province, China, in September 2020. The larvae were disinfected with 75% ethanol and then reared in an incubator in the dark (26 ± 2 °C, RH = 70 ± 5%) with an artificial diet [46]. 

The recipe of the artificial diet was according to Chen et al. [46], which consisted of 100 g pine sawdust, 40 g agar, 20 g sucrose, 12.5 g yeast extract, 2 g sodium benzoate, 1 g potassium sorbate, 10 mL 0.5 M sulfuric acid, 25 g wheat germ powder, 1.5 g cholesterol, 4 g ascorbic acid, 20 g casein, 1 g choline chloride, and 800 mL water. 

The 4th-instar larvae, which have relatively higher tolerance to α-pinene [17], were used in our study. After starvation for 24 h, the larvae were divided into four groups and fed an artificial diet containing α-pinene (Aladdin, Shanghai, China) of different concentrations (2.8, 5.6, and 11.2 mg/g), which were named the low-, medium- and high-dose groups, respectively. Larvae fed a diet containing no α-pinene were used as control. All treatment groups were used for experiments unless otherwise stated. 

### 4.2. Determination of Hydrogen Peroxide, MDA, and Activity of GSTs

The larvae were dissected to take intestinal samples after treatment for different durations (24, 48, and 72 h). The samples were ground in an ice bath after accurately weighing them. Subsequently, the homogenate was centrifuged at 600× *g* for 10 min, and the supernatant was used for assays. 

Determination of hydrogen peroxide was performed with a hydrogen peroxide assay kit (Nanjing Jiancheng Bioengineering Institute, Nanjing, China) according to the manufacturer’s instructions. In brief, the supernatant was mixed with molybdic acid solution. The complex generated by hydrogen peroxide and molybdic acid has its maximum absorption peak at 405 nm. Thus, the hydrogen peroxide content was calculated by the absorbance at 405 nm and defined as the amount of produced complex per gram of tissue homogenate protein. 

MDA was determined using a malondialdehyde (MDA) assay kit (TBA method) (Nanjing Jiancheng Bioengineering Institute, Nanjing, China) according to the manufacturer’s instructions. Briefly, the supernatant was mixed with thiobarbituric acid (TBA) and trichloroacetic acid (TCA) and then heated in boiling water for 20 min at 95 °C. The sample solution was cooled and centrifuged at 1500× *g* for 10 min. Condensation products of MDA and TBA have their maximum absorption peak at 532 nm. Therefore, the MDA content was calculated using the absorbance at 532 nm and defined as the amount of condensation products per gram of tissue homogenate protein.

A GST activity assay was performed using a glutathione *S*-transferase (GST) activity assay kit (Sangon Biotech, Shanghai, China) according to the manufacturer’s instructions. To be brief, the supernatant was mixed with 1-chloro-2,4-dinitrobenzene (CDNB) and reducing glutathione solution (GSH). Absorbance at 340 nm was recorded at 30 s intervals to calculate the GST activity. Each milligram of tissue homogenate protein that catalyzes 1 µmol of CDNB binding to GSH per minute was defined as an enzyme activity unit. 

### 4.3. RNA Isolation and cDNA Synthesis

Larvae of the low-dose group were used for total RNA isolation with a TRNzol Universal kit (Tiangen^®^, Beijing, China) under the manufacturer’s instructions. RNA integrity was evaluated on 1% agarose gels and quantified with NanoDrop 2000 (Thermo Scientific, Pittsburg, PA, USA). Samples with high-quality bands were used for cDNA synthesis with an Evo M-MLV RT Mix Tracking Kit (Accurate Biology, Changsha, China). Concentration and quality of cDNA samples were determined with NanoDrop 2000. 

### 4.4. Amplification and Cloning of MaGSTe3

Based on the transcriptome database constructed in our previous study [17], the unique genes encoding the GSTs of the functional annotations were collected. The full-length open reading frame (ORF) of *MaGSTe3* was confirmed using SeqBuilder of DNAStar (DNASTAR, Madison, WI, USA). 

The synthesized cDNA from total RNA was used as the template for amplification with ApexHF HS DNA Polymerase FS (Accurate Biology, Changsha, China). The primers used for PCR reaction with complete ORF (Table A1) were designed using Primer 5.0. The reaction was performed in a 50 µL volume containing 3 µL cDNA, 1 µL of each primer (10 mM), 25 µL 2× ApexHF FS PCR Master Mix, and 20 µL nuclease-free water. The processes of amplification were 30 s of initial denaturation at 94 °C, a cycling protocol consisting of 35 cycles of denaturation at 98 °C for 10 s, annealing at Tm for 15 s, and extension at 72 °C for 5 s. 

The PCR products were recovered using a SteadyPure Agarose Gel DNA Purification Kit (Accurate Biology, Changsha, China) and subsequently ligated to circular plasmids using a 5 min TA/Blunt-Zero Cloning Kit (Vazyme, Nanjing, China). The plasmids were transformed into *Escherichia coli* DHα5 (Vazyme, Nanjing, China). Single colonies were shaking cultured on Luria–Bertani (LB) medium for 1 h at 37 °C at 200 rpm. Positive bacterial fluid was selected and sent to Sangon Biotech (Shanghai) Co., Ltd. (Shanghai, China) for sequencing.

### 4.5. Sequence and Phylogenetic Analysis of MaGSTe3

The theoretical isoelectric points and the molecular weight of MaGSTe3 were predicted using ExPASy (https://web.expasy.org/, accessed on 4 March 2022). The subcellular localization was predicted with the website (http://cello.life.nctu.edu.tw/, accessed on 4 March 2022). The conserved domain of MaGSTe3 was predicted with the Conserved Domain Database of NCBI (https://www.ncbi.nlm.nih.gov/cdd/, accessed on 4 March 2022). The three-dimensional structure of MaGSTe3 was predicted through SWISS-MODEL (https://swissmodel.expasy.org/, accessed on 4 March 2022) [47].

The obtained sequences were aligned with 64 insect GST sequences collected from Uniprot (https://www.uniprot.org/, accessed on 4 March 2022) and NCBI (https://www.ncbi.nlm.nih.gov/, accessed on 4 March 2022) using DNAMAN 8.0 (Lynnon Biosoft, San Ramon, CA, USA). Then, a phylogenetic tree was constructed by the maximum likelihood method using Mega 7.0 (https://www.megasoftware.net/download_form, accessed on 1 May 2021), and the bootstrap value was set to 1000. 

### 4.6. qRT-PCR

The cDNA used for qRT-PCR was obtained with the methods mentioned above. Primers (Table A2) were designed with Primer-BLAST (https://www.ncbi.nlm.nih. gov/tools/primer-blast/, accessed on 4 March 2022). To estimate the qPCR efficiency and validation, the cDNA was diluted to 200 ng/μL and then diluted to five gradients (1.0, 10^−1^, 10^−2^, 10^−3^, and 10^−4^). The standard curve was established after the amplification of cDNA of different dilutions and their initial concentrations. Primers with R^2^ > 0.98 and 100 ± 10% efficiency were used for subsequent experiments.

The qRT-PCR reactions were carried with the ABI 7900HT Real-Time PCR System using a Hieff qPCR SYBE Green Master Mix (Yeasen, Shanghai, China). The reaction was performed in a 20 μL volume containing 2 μL cDNA templates, 0.4 μL of each primer (10 μM), 10 μL of Hieff qPCR SYBE Green Master Mix, and 7.2 μL of nuclease-free water. The thermal cycle conditions were 5 min of initial denaturation at 95 °C, a cycling protocol consisting of 40 cycles of denaturation at 95 °C for 10 s, and annealing at 60 °C for 40 s. The ribosomal protein L10 (*RPL10*) gene of *M. alternatus* was used as the reference gene. The assay was performed in triplicate and repeated three times. The expression level was quantified using the 2^−ΔΔCt^ method.

### 4.7. Prokaryotic Expression and Purification of Recombinant MaGSTe3 Protein

The entire coding sequence of *MaGSTe3* was amplified with primers containing the specific sequences of the vector. The amplified fragments were then inserted into pET-28a (+) stored in our laboratory and then transformed into *E. coli* BL21 (DE3) (Vazyme, Nanjing, China). The cells were shaking-cultured under the condition of 37 °C and 200 rpm in LB liquid medium with kanamycin. When the optical density at 600 nm (OD_600_) reached the range of 0.4 to 0.6, 600 μL 500mM isopropyl β-d-1-thiogalactopyranoside (IPTG) was added to induce the expression of recombinant proteins at 20 °C at 200 rpm for 18 h. The harvested cells were sonicated and then centrifuged at 13,500× *g* and 4 °C for 25 min. The supernatant was purified using nickel–nitrilotriacetic acid (Ni–NTA) resin. The recombinant GST protein was evaluated by sodium dodecyl sulfate polyacrylamide gel electrophoresis (SDS–PAGE). Purified protein was dialyzed with three dialysates (Table A3) in a dialysis bag (25 × 18 cm, 15 kDa retention molecular weight), frozen for 6 h, and then diluted to 1 mg/mL. The protein concentrations were determined using a total protein quantitative assay kit (Jiancheng, Nanjing, China).

### 4.8. Enzyme Activity Assays of the Recombinant MaGSTe3

To determine the activity of recombinant MaGSTe3, 1-chloro-2,4-dinitrobenzene (CDNB) was used as the substrate. All reactions were performed with a total reaction volume of 200 μL containing 2 μL protein, 2 μL GSH, 2 μL CNDB, and 194 μL 100mM PBS. 

The kinetic parameters of recombinant MaGSTe3 were determined by various concentrations (0–400 mM) of CDNB substrate. The boiled inactivated recombinant MaGSTe3 was used as control, and each test was repeated three times independently.

Special activity (SA) was measured to construct an enzyme kinetic curve. OD_340_ was measured at 30 s intervals for 5 min for calculation of SA. The SA of GST was expressed as per mole of CDNB conjugated with GSH per minute per milligram of protein using the extinction coefficient of the resulting 2,4-dinitrophenyl-glutathione, which is about 9.6 mM/cm. The enzyme kinetic curve was fitted using the Michaelis–Menten equation by Origin 2021 (OriginLab, Northampton, MA, USA).

The optimum temperature of MaGSTe3 was assayed by incubating the recombinant protein in PBS (pH 7.4) at a 10–60 °C temperature gradient for 30 min. The optimum pH for MaGSTe3 activity was investigated with different PBS with a pH range of 4.0 to 11.0. The SA was measured, and the maximum SA of each experiment was considered as 100% of the relative enzyme activity. 

### 4.9. Disk Diffusion Assay

A disk diffusion assay was performed following Burmeister et al. [48] with some modification. The *E. coli* BL21 (DE3) culture containing overexpressed *MaGSTe3* was plated onto LB agar plates with kanamycin. Each plate contained about 5 × 10^8^ cells, which were measured by an Auto Colony Counter MF2 (SHINESO, Hangzhou, China). After incubation at 37 °C for 1 h, filter discs 6 mm in diameter soaked with various concentrations of cumene hydroperoxide (CHP, 0, 25, 50, 100, and 200 mM) were placed on the plates and then incubated at 37 °C for 24 h. The bacteriostatic rings were analyzed using Auto Colony Counter MF2. 

### 4.10. RNA Interference

To obtain dsRNA fragments, pairs of primers (Table A5) were designed based on the ORF of *MaGSTe3* with Primer 5.0, and the T7 promoter (TTAATACGACTCACTATAGGG) was added to both ends of primers using the T7 RiboMAXTM Express RNAi System (Promega, Madison, WI, USA). A PCR reaction was performed for DNA used as a dsRNA template with the method described above. After recovery and purification, the products were transcribed into ds*MaGSTe3* and subsequently injected into the 4th-instar larvae. The RNAi-treated larvae were treated with a diet containing 5.6 mg/g α-pinene, and their guts were taken after treatment for 24 h for hydrogen peroxide MDA and GST activity determination. Meanwhile, the RNAs were extracted for qRT-PCR using the above-mentioned methods.

### 4.11. Statistical Analysis

The data were analyzed using SPSS 25.0 (IBM, Armonk, NY, USA). The differences between treatment and control groups were analyzed by one-way ANOVA with a significance level of *p* < 0.05. The values were presented as the mean value ± standard deviation (SD) of three independent experiments.

## 5. Conclusions

Our results suggested that MaGSTe3 takes part in α-pinene adaptation in *M. alternatus* larvae through protecting tissues from oxidative stress but does not play a major role.

## Data Availability

The data that support the findings of this study are available from the corresponding author upon reasonable request.

## Appendix B

**Table A1:** Primer used in the GST genes cloning.

Prime Name	Primer Sequence (5′→3′)
MaGSTe3-F	ATGACGGTTATAGTGTATGGGAC
MaGSTe3-R	TTAAGCCAGTTTGCTCTTAAACGCTG

**Table A2:** Primer used in the GSTs qRT-PCR.

Prime Name	Primer Sequence (5′→3′)
RPL-10-qF	GACAAACGTTTCAGCGGAAC
RPL-10-qR	TGCTGTTGATCGCCCAAAAC
MaGSTe3-qF	CAGAAGAGGGCCGTGGTTA
MaGSTe3-qR	CAGCAGCGTAAGTGCTTCTT

**Table A3:** Dialysates used for dialysis of purified protein MaGSTe3 protein.

Dialysates Name	Components
Dialysates I	40 mM PBS (pH = 7.4), 200 mM NaCl, 200 mM imidazole
Dialysates II	20 mM PBS (pH = 7.4), 100 mM NaCl, 10 mM imidazole
Dialysates III	sterile water

Note: PBS refers to phosphate buffer solution.

**Table A4:** The basic characteristics of *MaGSTe3*.

Gene Name	Length	Mw	pI	BLASTX Best Hit				Subcellular Location
	(bp)	(kDa)		Species	Acc. No.	E-Value	Identity	
							(%)	
*MaGSTe3*	648	23.58	5.72	*Anoplophora glabripennis*	XP_018564047.1	2 × 10^−9^	99	Cytoplasmic

**Table A5:** Primers for RNA interference of *MaGSTe3*.

Prime Name	Primer Sequence (5′→3′)
ds-MaGSTe3-T7-F	taatacgactcactatagggAGTGAACACTATGGCTGGGG
ds-MaGSTe3-T7-R	taatacgactcactatagggGACGCGTAGTAAGGCAAAGC
ds-GFP-T7-F	ggatcctaatacgactcactataggATGAGTAAAGGAGAAGAACTTTTC
ds-GFP-T7-R	ggatcctaatacgactcactataggTTTGTATAGTTCATCCATGCCAT
ds-MaGSTe3-q-F	ACCAGTGCCACTACAACAATG
ds-MaGSTe3-q-R	GTTCCTCTGACGACTGGACT
